# Combined Mass Spectrometry-Based Metabolite Profiling of Different Pigmented Rice (*Oryza sativa* L.) Seeds and Correlation with Antioxidant Activities

**DOI:** 10.3390/molecules191015673

**Published:** 2014-09-29

**Authors:** Ga Ryun Kim, Eun Sung Jung, Sarah Lee, Sun-Hyung Lim, Sun-Hwa Ha, Choong Hwan Lee

**Affiliations:** 1 Department of Bioscience and Biotechnology, Konkuk University, Seoul 143-701, Korea; 2 National Academy of Agricultural Science, Rural Development Administration, Suwon 441-707, Korea; 3 Department of Genetic Engineering and Crop Biotech Institute, College of Life Sciences, Kyung Hee University, Suwon 446-701, Korea

**Keywords:** rice seed (*Oryza sativa* L.), UPLC-Q-TOF-MS, GC-TOF-MS, metabolite profiling, antioxidant activity

## Abstract

Nine varieties of pigmented rice (*Oryza sativa* L.) seeds that were black, red, or white were used to perform metabolite profiling by using ultra-performance liquid chromatography-quadrupole-time-of-flight mass spectrometry (UPLC-Q-TOF-MS) and gas chromatography (GC) TOF-MS, to measure antioxidant activities. Clear grouping patterns determined by the color of the rice seeds were identified in principle component analysis (PCA) derived from UPLC-Q-TOF-MS. Cyanidin-3-glucoside, peonidin-3-glucoside, proanthocyanidin dimer, proanthocyanidin trimer, apigenin-6-*C*-glugosyl-8-*C*-arabiboside, tricin-*O*-rhamnoside-*O*-hexoside, and lipids were identified as significantly different secondary metabolites. In PCA score plots derived from GC-TOF-MS, Jakwangdo (JKD) and Ilpoom (IP) species were discriminated from the other rice seeds by PC1 and PC2. Valine, phenylalanine, adenosine, pyruvate, nicotinic acid, succinic acid, maleic acid, malonic acid, gluconic acid, xylose, fructose, glucose, maltose, and *myo*-inositol were significantly different primary metabolites in JKD species, while GABA, asparagine, xylitol, and sucrose were significantly distributed in IP species. Analysis of antioxidant activities revealed that black and red rice seeds had higher activity than white rice seeds. Cyanidin-3-glucoside, peonidin-3-glucoside, proanthocyanidin dimers, proanthocyanidin trimers, and catechin were highly correlated with antioxidant activities, and were more plentiful in black and red rice seeds. These results are expected to provide valuable information that could help improve and develop rice-breeding techniques.

## 1. Introduction

Rice (*Oryza sativa* L.) is a staple cereal food, particularly in Asia, which accounts for 95% of global rice production and consumption [1]. Rice can be divided into two types: pigmented and non-pigmented. Non-pigmented rice is consumed by approximately 85% of the world’s population, while pigmented rice has been mostly consumed as a specialty in China, Japan, and Korea for its flavor and health benefits [2]. Black rice is frequently mixed with white rice during cooking to enhance flavor and nutritional value, and red rice is often used to make bread, ice cream, and liquor [3,4]. Recently, pigmented rice has attracted interest because it was reported to have potent antioxidant activities and a higher content of phenolic components [5]. The major active components of pigmented rice seeds are the anthocyanins and proanthocyanidins [6]. Anthocyanins possess antioxidant [7], anti-inflammatory [8], antitumor [9], and hypoglycemic effects [10]. Compared with anthocyanidins, proanthocyanidins, which can found in some cereals, legume seeds, and various fruits, have superior antioxidant activities [11].

Recently, metabolite profiling has been used as a valuable tool in many plant studies, including plant-derived food analysis [12], crop metabolite profiling [13], plant metabolite analysis [14,15], and in the development of plant-derived medicines [16]. The use of gas chromatography coupled with time-of-flight mass spectrometry (GC-TOF-MS) for metabolite analysis offers a number of advantages, including high reproducibility, rapid scan times, high sample throughput, and peak deconvolution (*i.e.*, the capability to resolve overlapping peaks) [17]. The use of ultra-performance liquid chromatography-quadrupole-time-of-flight-mass spectrometry (UPLC-Q-TOF-MS) for metabolite analysis also offers many advantages, including accurate mass measurement, high resolution, and compound structure prediction [18].

Previously, rice metabolites have been extensively studied [19,20,21]. Gas chromatography–mass spectrometry (GC-MS) based metabolite analysis revealed differences in sugars, sugar alcohols, organic acids, amino acids, and fatty acid methyl esters among colored rice grains and also in the germination process [19,22]. Liquid chromatography–mass spectrometry (LC-MS) based targeted metabolite analysis of anthocyanin, carotenoid, and flavonoid compounds has also been performed [13,19,20,21,22,23]. In the case of Korean rice seeds, several studies have been reported. In particular, Park *et al.* and Kim *et al.* intensively revealed metabolites in a variety of Korean rice seeds including, Heugkwang (HK), Heugnam (HN), Heugjinju (HJJ), Hongjinju (HoJJ), Jeogjinju (JJJ), Hwaseong (HS), and Ilpoom (IP) cultivars by using GC-TOF-MS [21,24]. However, one of the Korean rice species Jakwangdo (JKD) has not been well studied, especially by using a metabolomics approach. Furthermore, all of these previous studies focused only on target metabolite analysis, or only the primary metabolites of rice seed. To the best of our knowledge, no previous metabolomics studies have used both GC-TOF-MS and UPLC-Q-TOF-MS, to compare overall metabolite differences between different Korean cultivars of rice seeds. In the present study, we performed metabolite profiling of nine different Korean pigmented rice (*O. sativa*) seeds by UPLC-Q-TOF-MS and GC-TOF-MS. In addition, we selected significantly different metabolites among different colors of rice seeds, and used them to perform a correlation analysis with the antioxidant activities.

## 2. Results and Discussion

### 2.1. Metabolite Profiling of Different Pigmented Rice Seeds by UPLC-Q-TOF-MS

**Figure 1:**
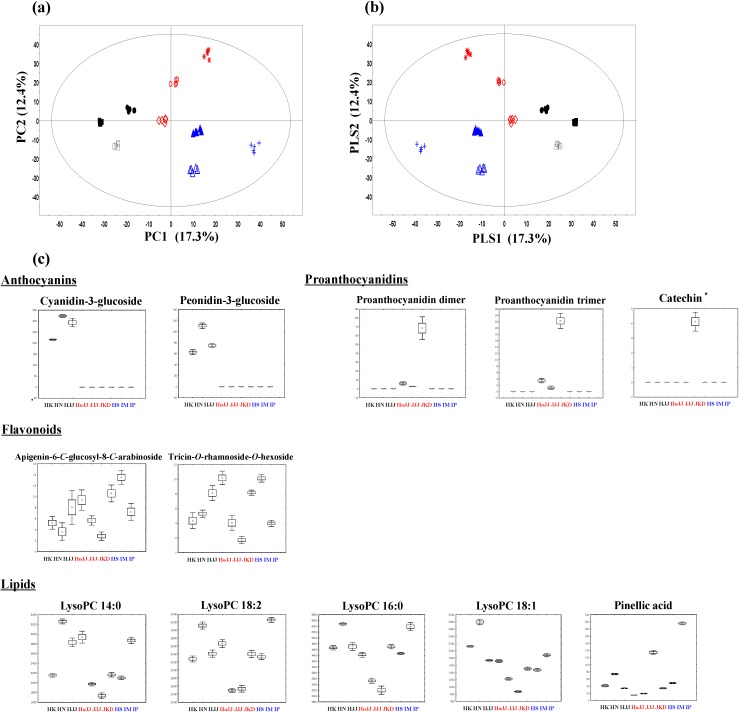
The principal component analysis (PCA) score plots (**a**), partial least-square discriminant analysis (PLS-DA) score plots (**b**), and box-whisker plots (**c**) of nine different varieties of rice seeds analyzed by UPLC-Q-TOF-MS. Black rice seed: ● HK (Heugkwang), □ HN (Heugnam), ■ HJJ (Heugjinju); Red rice seed: 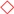 HoJJ (Hongjinju), 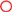 JJJ (Jeogjinju), 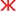 JKD (Jakwangdo); White rice seed: 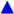 HS (Hwaseong), 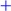 IM (Ilmi), 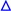 IP (Ilpoom). * Target identified.

Seeds from nine species of rice were analyzed by UPLC-Q-TOF-MS, and multivariate analysis was performed. Using principle component analysis (PCA), clear color grouping distributions can be seen on the score plot (Figure 1a). Black color rice seeds, including HK, HN, and HJJ, and white color rice seeds, including HS, IM, and IP, were clearly distinguished by PC 1 (17.3%). Red color rice seeds were separated from black and white color rice seeds by PC 2 (12.4%). Similar formations could be seen in the partial least squares discriminant analysis (PLS-DA) (Figure 1b). Eleven secondary metabolites, such as cyanidin-3-glucoside, peonidin-3-glucoside, proanthocyanidin dimer, proanthocyanidin trimer, apigenin-6-c-glucosyl-8-c-arabionoside, tricin-O-rhamnoside-O-hexoside, pinellic acid, lysoPC 14:0, lysoPC 18:2, lysoPC 16:0, and lysoPC 18:1, were identified as being significantly different among the nine varieties of rice seeds. Assignment of metabolites was carried out using high-resolution mass data (i-fit, ppm), MS^n^ fragment patterns, UV absorbance value, standard compounds, and references (Supplementary data, Table 1S). As shown in Figure 1c, relative contents of secondary metabolites among the nine varieties of rice seeds are shown by box-whisker plots. Cyanidin-3-glucoside and peonidin-3-glucoside, which are anthocyanin compounds, were detected only in black rice seeds. On the other hand, proanthocyanidin dimers and proanthocyanidin trimers, which are proanthocyanidin compounds, were only detected in red rice seeds. Usually, the anthocyanin contents were high in black rice seeds, and proanthocyanidin contents were high in red rice seeds. Finocchiaro *et al.* also suggested a similar distribution pattern of anthocyanin and proanthocyanidin in black and red rice seeds, respectively [25]. However, in our study anthocyanin compounds were only detected in black rice seeds and not in red rice seeds. Similarly, proanthocyanidin compounds were only detected in red rice seeds and were not detected in black rice seeds. However, our results are consistent with those of other studies that were unable to detect anthocyanin compounds in HoJJ and JJJ seeds [20]. Catechin, which is known as a precursor [26] of proanthocyanidins showed high distribution in JKD. In case of flavonoid compounds, tricin-*O*-rhamnoside-*O*-hexoside, and apigenin-6-*C*-glucosyl-8-*C*-arabionoside showed relatively high content in white rice seeds. These compounds are well known in white rice seeds [27,28,29]. In the case of lipids, lysoPCs are one of the main phospholipids present in rice and were found to have similar distribution patterns among rice seeds of the same color. However, there was some variability in distribution patterns between rice seeds of different colors. Pinellic acid has been isolated from rice cultivars in other studies [30], and a relatively high content of pinellic acid was found in JKD and IP seeds. Consequently, the color-specific patterns identified on the PCA score plot were mainly attributed to color-related metabolites anthocyanins and their precursors, proanthocyanidins and flavonoids.

### 2.2. Metabolite Profiling of Different Pigmented Rice Seeds by GC-TOF-MS

**Figure 2:**
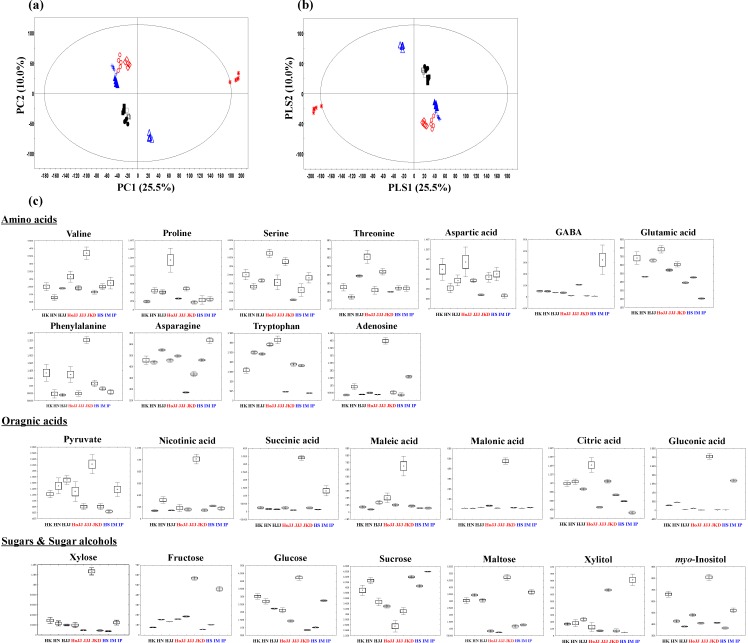
The principal component analysis (PCA) score plots (**a**), partial least-square discriminant analysis (PLS-DA) score plots (**b**), and box-whisker plots (**c**) of nine different varieties of rice seeds analyzed by UPLC-Q-TOF-MS. Black rice seed: ● HK (Heugkwang), □ HN (Heugnam), ■ HJJ (Heugjinju); Red rice seed: 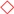 HoJJ (Hongjinju), 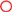 JJJ (Jeogjinju), 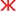 JKD (Jakwangdo); White rice seed: 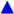 HS (Hwaseong), 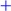 IM (Ilmi), 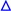 IP (Ilpoom).

To identify different primary metabolites between nine species of rice seeds, we performed GC-TOF-MS analysis, which we combined with multivariate analysis. According to the PCA plot (Figure 2a), JKD and IP species were distinguished from other species by PC1 (25.5%). Along with PC2 (10.0%), red rice (JKD, HoJJ, and JJJ) and white rice (IM and HS) were separated from white rice (IP) and black rice (HK, HN, and HJJ). On the basis of standard compounds, we identified a total of a 25 metabolites including 11 amino acids, seven sugars and sugar alcohols, and seven organic acids, as significantly different among nine rice seeds using VIP values (VIP > 0.7) and *p* values (*p* < 0.05). The retention times and MS fragment ions of these metabolites are summarized in Supplementary data, Table S2. The 25 identified metabolites from nine different varieties of rice seeds are presented by box-whisker plots (Figure 2c). Among them, glucose, sucrose, glutamic acid, and aspartic acid influence taste and function of the rice seeds, in particular, these metabolites directly affect the sweetness and palatability of cooked rice [31,32]. Valine, phenylalanine, adenosine, nicotinic acid, succinic acid, maleic acid, malonic acid, gluconic acid, xylose, fructose, glucose, *myo*-inositol, and maltose showed higher distribution in JKD species. The IP species-specific compounds were GABA, asparagine, sucrose, and xylitol. Functionally, GABA has been shown to suppress blood pressure and improve sleeplessness [33], and nicotinic acid has a lipid regulating effect [34]. Phenylalanine, which showed high contents in JKD, is a precursor of catechin and proanthocyanidin in the biosynthesis of phenylpropanoid compounds [26,35].

### 2.3. Correlation between Rice Seed Metabolites and Antioxidant Activities

**Figure 3:**
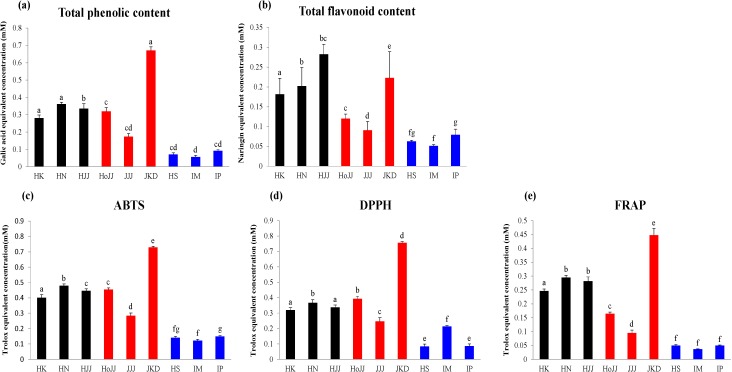
Total flavonoid contents (**a**), total phenolic contents (**b**), and antioxidant activity assays, ABTS (**c**), DPPH (**d**), and FRAP (**e**) of nine different varieties of rice seeds. Values represent averages of triplicate measurements. Different letters indicate significantly different values, according to Duncan’s multiple range test.

**Figure 4:**
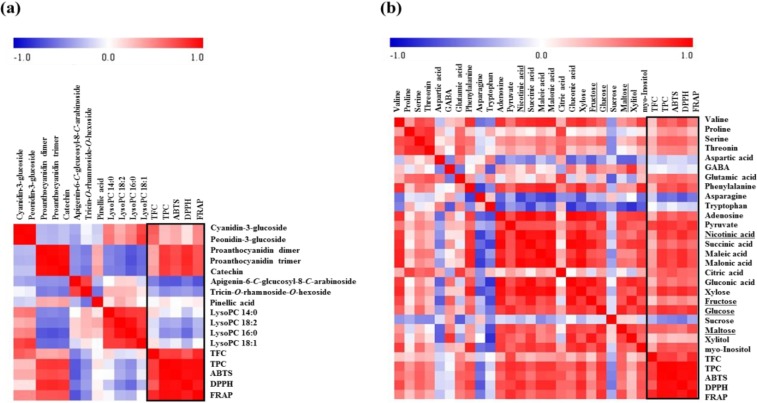
Correlation map of metabolites analyzed by UPLC-Q-TOF-MS and antioxidant activities (**a**) and correlation map of metabolites analyzed by GC-TOF-MS and antioxidant activities (**b**). Each square indicates *r* (Pearson’s correlation coefficient values for a pair of metabolites or antioxidant activities). The red color represents positive (0 < *r* < 1) correlation and blue color represents negative (−1 < *r* < 0) correlation.

To compare antioxidant activities between rice seeds, we measured antioxidant activity by using the following assays: ABTS, DPPH, and FRAP, TFC, and TPC (Figure 3). According to the results of the antioxidant activity assays, black and red rice seeds showed higher antioxidant activities than white rice seeds. In particular, one of the red seeds, JKD, showed the highest antioxidant activity. Results of TFC, showed the following order of antioxidant activities: black > red > white rice seeds. In TPC, the black and red rice seeds showed high total phenolic content compared with that in the white rice seeds, especially, the JKD species, which had the highest total phenolic contents. According to other studies, TPC showed a positive correlation with antioxidant activity (ABTS, DPPH, and FRAP) [36]. Anthocyanin and proanthocyanidin compounds possess antioxidant activities [2,6]. In particular, proanthocyanidin compounds are known to have considerable antioxidant activities, even higher than those possessed by anthocyanin compounds [11]. As shown in Figure 1c, cyanidin-3-glucoside and peonidin-3-glucoside were only detected in black rice seeds in the following order HN > HJJ > HK. Proanthocyanidin dimers and trimers were only detected in red rice seeds and were found to be particularly high in JKD species. Catechin, which is known as an antioxidant, was detected only in JKD seeds [36]. JKD seeds were found to possess remarkably high contents of TPC, proanthocyanidin dimers, proanthocyanidin trimers, and catechin, which correlate with their antioxidant activities. To visualize the correlation between metabolites and antioxidant activities, we produced a correlation map using Pearson’s correlation coefficient (Figure 4). As shown in Figure 4a, TPC and TFC showed a high positive correlation with antioxidant activities. Five secondary metabolites, cyanidin-3-glucoside, peonidin-3-glucoside, proanthocyanidin dimer, proanthocyanidin trimer, and catechin, showed positive correlation with antioxidant activities, while apigenin-6-*C*-glucosyl-8-*C*-arabionoside, tricin-*O*-rhamnoside-*O*-hexoside, and lipids showed negative correlation with antioxidant activities. Cyanidin-3-glucoside, peonidin-3-glucoside, proanthocyanidin dimer, proanthocyanidin trimer, and catechin have been reported to have high antioxidant activities in several studies [2,6]. In Figure 4b, correlation maps between metabolites analyzed by GC-TOF-MS and antioxidant activities are shown. Some amino acids (valine, proline, serine, threonine, glutamic acid, phenylalanine, and adenosine), organic acids (pyruvate, nicotinic acid, succinic acid, maleic acid, malonic acid, citric acid, gluconic acid), and sugars and sugar alcohols (xylose, fructose, glucose, *myo*-inositol, maltose) showed positive correlations with each other and also positively correlated with antioxidant activities (Figure 4b). Among these, fructose, glucose, and maltose were found to have measurable free radical scavenging activities by different methods in other studies, which can explain the antioxidant potentials of these compounds [37]. Furthermore, nicotinic acid has previously been reported to have antioxidant activities [38]. We suspected that JKD species would possess high antioxidant activities because of their high content of antioxidant compounds. This information will be useful for functional breeding in future.

## 3. Experimental Section

### 3.1. Chemicals and Reagents

Water, methanol, and chloroform were purchased from Fisher Scientific (Pittsburgh, PA, USA). Methoxyamine hydrochloride, *N*-methyl-*N*-(trimethylsilyl) (MSTFA), pyridine, formic acid, 6-hydroxy-2,5,7,8-tetramethylchromane-2-carboxylic acid (Trolox), 1,1-diphenyl-2-picrylhydrazyl (DPPH), 2,2'-azinobis(3-ethylbenzothiazoline-6-sulfonic acid) diammonium salt (ABTS), 2,4,6,-tris(2-pyridyl)-s-triazine (TPTZ), Iron(III) chloride hexahydrate, acetic acid, sodium acetate, hydrochloride, Folin—Ciocalteu’s phenol reagent, sodium carbonate, diethylene glycol, potassium persulfate, and standard compounds were obtained from Sigma Chemical Co. (St. Louis, MO, USA).

### 3.2. Plant Materials

Rice (*Oryza sativa* L.) seeds were obtained from the Agricultural Genetic Resources Center at the National Academy of Agricultural Science (Suwon, Korea) and from the division of Rice Research at the National Institute of Crop Science (Suwon, Korea). Nine differently pigmented rice cultivars were used in this study and were categorized according to their pericarp color as follows; black: heugkwang (HK), heugnam (HN), and heugjinju (HJJ); red: hongjinju (HoJJ), jeogjinju (JJJ) and jakwangdo (JKD); and white: hwaseong (HS), ilmi (IM), and ilpoom (IP). Rice seeds were stored at 4 °C in a cold room before analysis.

### 3.3. Sample Preparation

The seeds were manually dehulled with a wooden rice dehuller and ground to a powder by using a mortar and pestle. The milled rice powders were stored at −80 °C until further use. Rice powders (100 mg) were extracted in 1 mL of mixed solvent (methanol/water/chloroform = 2.5:1:1 by vol.) by using a thermomixer (Eppendorf AG, Germany) at 700 rpm for 30 min at 37 °C. After the addition of 0.4 mL of water, samples were centrifuged for 10 min at 10,000 rpm at 25 °C. The polar phase was collected into a new tube and then dried in a speed vacuum concentrator (Biotron, Seoul, Korea). Prior to GC-TOF-MS analysis, methoximation and silylation were performed. The methyloxime derivative was added by dissolving the dried extracts in 50 μL of methoxyamine hydrochloride (20 mg/mL) in pyridine, and shaking for 90 min at 30 °C. After methoximation, the samples were silylated with 50 μL of MSTFA for 30 min at 37 °C. Prior to UPLC-Q-TOF-MS analysis, dried samples were resuspended in methanol.

### 3.4. GC-TOF-MS Analysis

We performed GC-TOF-MS by using an Agilent 7890A gas chromatograph system (Agilent, Atlanta, GA, USA) equipped with an Agilent 7693 autosampler, coupled to a Pegasus HT TOF mass spectrometer (Leco Corp., St. Joseph, MI, USA). We used an Rtx-5MS fused-silica capillary column (i.d. 30 m × 0.25 mm, 0.25 μm particle size; Restek Corp., Bellefonte, PA, USA) with helium gas at a constant flow rate of 1.0 mL/min. The temperature program was as follows: initial temperature of 75 °C for 2 min; followed by an increase to 300 °C at 15 °C/min; and a final 3-min hold at 300 °C. The injector and transfer line temperatures were 250 °C and 240 °C, respectively. The scanned mass range was 50–1000 *m/z*, and the electron energy was set at −70 eV.

### 3.5. UPLC-Q-TOF-MS and LC-IT-MS/MS Analysis

We performed UPLC by using a Waters Acquity UPLC system (Water Corp., Milford, MA, USA), equipped with a binary solvent delivery system, an autosampler, and a UV detector. The UPLC system contained an Acquity BEH C18 (i.d. 100 mm × 2.1 mm, 1.7-μm particle size; Waters Crop). The mobile phases, A and B, were composed of 0.1% (v/v) formic acid in water, and 0.1% (v/v) formic acid in acetonitrile, respectively. The initial condition was 5% solvent B for 1 min. The linear gradient was then increased from 5% solvent B to 100% solvent B over 9 min. The injection volume was 5 μL, and the flow rate was 0.3 mL/min. The Waters Q-TOF Premier (Micromass MS Technologies, Manchester, UK) was operated on both negative and positive ion modes with a mass range of 100–1000 *m/z*. The capillary and cone voltages were set to 3.0 kV and 30 V, respectively. The source temperature was set at 100 °C; the desolvation gas flow was 700 L/h at a temperature of 300 °C; the collision gas flow was 0.3 mL/min; and the collision energy was set at 5 eV. The V mode was used for mass spectrometry, and data were collected in the centroid mode with a scan accumulation time of 0.2 s. Leucine enkephalin was used as reference lock mass (*m/z* 554.2615 [−] and 556.2771 [+], 10 μL/min), using independent LockSpray interference.

We performed (LC-ESI-IT-MS/MS) by using a 212-LC binary gradient solvent delivery pump, a ProStar™ 410 autosampler, and a ProStar™ 335 photodiode array detector, coupled to a Varian 500-MS ion trap-mass spectrometer equipped with an electrospray interface (Varian Tech., Palo Alto, CA, USA). We separated 10 μL of sample on a PurSuit XRs C18 column (i.d. 100 mm × 2.0 mm; 3 μm particle size; Varian tech) with water (mobile A) and acetonitrile (mobile B) containing 0.1% formic acid at a flow rate of 0.2 mL/min. For sample analysis, the initial solvent condition was 10% solvent B; the gradient was then gradually increased from 10% solvent B to 100% solvent B over 25 min. We used a photodiode array set at 200–600 nm for detection and managed by PolyView 2000 (version 6.9) (Varian, Walnut Creek, CA). We performed tandem MS analysis using scan type turbo data-dependent scanning (DDS) through an *m/z* range of 50–2000. The parameters of negative and positive ion modes applied for each sample were as follows: drying temperature, 300 °C; needle voltage, 5 kV; drying gas pressure (nitrogen), 10 psi; and nebulizer gas pressure (air), 35 psi. Data were recorded in continuous mode within a mass scan mode of 15,000/s. The mass scan average was set at three microscans (2.19 s/scan).

### 3.6. Determination of Antioxidant Activities Using ABTS, DPPH, and FRAP Assays

We performed ABTS assay by following the method described by Re *et al.* with some modifications [39]. Briefly, we mixed 7 mM ABTS solution with 2.45 mM potassium persulfate solution, and stored the mixture for 12 h in the dark at room temperature. The solution was diluted until the absorbance measured at 734 nm reached 0.7 ± 0.03 by using a microplate reader. Next, we mixed 20 μL of each rice extract with 180 μL of diluted ABTS solution in 96-well plates, and incubated for 6 min in the dark. We then measured the absorbance at 734 nm using a microplate reader (BioTek Instruments, Winooski, VT, USA).

We performed the DPPH assay according to the method of Dietz *et al.* with some modifications [40]. We mixed 20 μL of each rice extract with 180 μL of DPPH ethanol solution (0.2 mM) in 96-well plates, and incubated for 20 min at room temperature. We then measured the absorbance at 515 nm, using a microplate reader.

We performed the ferric ion reducing antioxidant power (FRAP) assay according to the method of Benzie *et al.* with some modifications [41]. The FRAP reagent was freshly prepared by mixing acetate buffer (pH 3.6), 10 mM TPTZ (in 40 mM HCl solution), and 20 mM FeCl3·6H2O (in distilled water) at a ratio of 10:1:1. Next, we mixed 10 μL of rice extracts with 300 μL of FRAP solution in 96-well plates and incubated for 6 min at 37 °C. Then, we measured the absorbance at 570 nm using a microplate reader. All of the experiments were performed in triplicate, and all of the results are presented as the trolox equivalent antioxidant capacity, with concentration ranges of 0.0156–2 mM. All of the assays were performed same rice extracts of mass spectrometry analysis, and the analysis was conducted at a working concentration of 50 ppm.

### 3.7. Determination of Total Phenolic Contents (TPC) and Total Flavonoid Contents (TFC)

Total phenolic contents were determined as described by Singleton *et al.* with some modifications [42]. Briefly, we mixed 20 μL of extract from each sample and 100 μL of 0.2 N Folin-Ciocalteu’s phenol reagents in 96-well plates. After 5 min reaction in the dark, 80 μL of 7.5% Na_2_CO_3_ solution (in distilled water) was added to the mixture, which was then incubated for 60 min at room temperature. The absorbance was measured at 750 nm by using a microplate reader. The results are presented as the gallic acid equivalent concentration (ppm), with concentration ranges of 31.25–500 ppm.

Total flavonoid contents were also measured. Briefly, 20 μL of extract from each sample, 20 μL of 1 N NaOH, and 180 μL of 90% diethylene glycol (in distilled water) was placed in 96-well plates and was mixed and incubated for 60 min at room temperature. The absorbance was measured at 405 nm by using a microplate reader. The results are presented as the naringin equivalent concentration (ppm). The standard solution concentration curve ranged from 15.625 to 200 ppm. All experiments were carried out in triplicate.

### 3.8. Data Processing and Multivariate Analysis

We processed the GC-TOF-MS raw data files with Chroma TOF software (version 4.44; Leco Ltd., St. Joseph, MI, USA), and the UPLC-Q-TOF-MS raw data files with MassLynx software (version 4.1; Waters Corp, Milford, MA, USA). Each of the software packages converted the raw data files into netCDF (*.cdf) format. After conversion, we processed the CDF files with the MetAlign software [43] to obtain peak detection, alignment, and retention time correction. The resulting data were exported to Excel files (Microsoft, Redmond, WA, USA), and multivariate statistical analysis was performed with SIMCA-P+ software (version 12.0; Umetrics, Umea, Sweden). The log-transformed MS data were mean-centered with unit variance scaling. We performed principal component analysis (PCA) and partial least-square discriminant analysis (PLS-DA) to compare metabolite differences between samples. We selected metabolites with variable importance in the projection (VIP) values of > 0.7, and a *p* value < 0.05 was considered significant. Significantly different metabolites were represented by box-whisker plots using Statistica, version 7.0 (StatSoft Inc., Tulsa, OK, USA). For the TFC, TPC analysis, and antioxidant activity tests (ABTS, DPPH, and FRAP) differences were tested by analysis of variance and Duncan’s multiple range tests using PASW Statistics 18 (SPSS Inc., Chicago, IL, USA). Pairwise correlations between metabolites and TFC, TPC, and antioxidant activities were calculated by Pearson’s correlation coefficient test using PASW Statistics 18 (SPSS, Chicago, IL, USA) and correlation map represented using MEV software version 4.8 (multiple array viewer) [44].

## 4. Conclusions

In conclusion, we performed unbiased metabolomics profiling of pigmented rice seeds using UPLC-Q-TOF-MS and GC-TOF-MS. A PCA score plot, analyzed by UPLC-Q-TOF-MS, was clarified by the color of rice seeds *viz.*, black, red, and white. Cyanidin-3-glucoside, peonidin-3-glucoside, proanthocyanidin dimers, proanthocyanidin trimers, and catechin exhibited color specific distributions across seed varieties, and were also found to have positive correlations with antioxidant activities. According to the PCA score plots derived from GC-TOF-MS, JKD and IP species were discriminated from other species of rice seed. Among significantly different primary metabolites, valine, phenylalanine, adenosine, pyruvate, nicotinic acid, succinic acid, maleic acid, malonic acid, gluconic acid, xylose, fructose, glucose, maltose, and *myo*-inositol were found in high concentrations in JKD species, while GABA, asparagine, and sucrose were highly concentrated in IP species. These include the antioxidants fructose, glucose, maltose, and nicotinic acid. We expected that JKD species, which are a red rice seed, would possess high antioxidant activities because of the high content of antioxidant compounds in this variety. This finding will be helpful for future functional breeding.

## Supplementary Materials

Supplementary materials can be accessed at: http://www.mdpi.com/1420-3049/19/10/15673/s1. List of tentatively identified secondary metabolites from different of rice seeds using UPLC-Q-TOF-MS (Table S1) and significantly different primary metabolites identified from different species of rice seeds extracts using GC-TOF-MS (Table S2).

## Supplementary Files

Supplementary File 1
