# Supplementary material for: Combined Mass Spectrometry-Based Metabolite Profiling of Different Pigmented Rice (Oryza sativa L.) Seeds and Correlation with Antioxidant Activities
Source: Molecules. 2014 Sep 29;19(10):15673–86. doi: 10.3390/molecules191015673 (PMC6271636; doi:10.3390/molecules191015673)
Supplement: Supplementary File 1 [file molecules-19-15673-s001.pdf]

Supplementary Materials

Table S1. List of tentatively identified secondary metabolites of nine different Korean species of rice seeds using UPLC-Q-TOF-MS.

Tentative Identification [a]	UPLC-Q-TOF-MS						LC-ESI-MS/MS			
	RT [b] (min)	Measured Mass (m/z)		Elemental Composition (Formular)	Δppm	iFit	MS^n Fragments (m/z)	Adduct	λ_{max}	Ref. [c]
Cyanidin-3-glucoside	3.11	447.09	449.108	$C_{21}H_{21}O_{11}$	−3.3	0.11	449 > 287 > 213 (+)	$[M+H]^+$	277, 515	std [d]
Peonidin-3-glucoside	3.35	461.108	463.127	$C_{22}H_{23}O_{11}$	2.6	0.72	463 > 301 > 286 (+)	$[M+H]^+$	276, 517	std
Pronathocyanidin dimer	3.1	577.133	579.153	$C_{30}H_{27}O_{12}$	5.2	1.96	579 > 453 > 301(+)	$[M+H]^+$	241, 278	[6,45]
Proanthocyanidin trimer	3.14	865.191	867.218	$C_{45}H_{39}O_{18}$	1.4	3.06	865 > 695 > 543 (−)	$[M-H]^-$	242, 277	[6,45]
Catechin *	3.23	289.071	291.093	$C_{15}H_{13}O_6$	2.1	0.84	289 > 253 > 203 (−)	$[M-H]^-$	241, 277	std
Apigenin-6-*C*-glucosyl-8-*C*-arabinoside	3.64	563.138	565.164	$C_{26}H_{27}O_{14}$	0.4	0.51	563 > 473 > 383 (−)	$[M-H]^-$	273, 308	[27,28]
Tricin-*O*-rhamnoside-*O*-hexoside	4.18	637.172	639.199	$C_{29}H_{35}O_{16}$	6.3	2.4	639 > 493 > 331 (+)	$[M+H]^+$	272, 313	[29]
Pinellic acid	5.82	329.233	353.232	$C_{18}H_{33}O_5$	−7.6	0.03	329 > 311,229,171 > 293 (−)	$[M-H]^-$	231, 313	[30]
LysoPC 14:0	7.71	512.297	468.312	$C_{23}H_{47}NO_9P$	2	1.08	512 > 452 > 227 (−)	$[M+FA-H]^-$	239, 264	[43,44]
LysoPC 18:2	8.28	564.334	520.343	$C_{27}H_{51}NO_9P$	−1.9	2.44	564 > 504 > 279 (−)	$[M+FA-H]^-$	239, 271	[43,44]
LysoPC 16:0	8.75	480.31	496.342	$C_{24}H_{51}NO_7P$	2	0.16	496 > 184 > 86 (+)	$[M+CH_3]^+$	241, 304	[43,44]
LysoPC 18:1	8.98	566.346	522.359	$C_{27}H_{53}NO_9P$	−3	0.18	566 > 506 > 281 (−)	$[M+FA-H]^-$	237, 273	[43,44]

[a] Identified metabolites based on VIP > 0.7, and a p-value < 0.05 from the PLS-DA model. [b] Retention time. [c] Reference. [d] Standard compound. * Target identified.

Table S2. Significantly different primary metabolites identified from nine different species of rice seeds extracts using GC-TOF-MS.

RT [a] (min)	Metabolites [b]	MS Fragment Ions (*m/z*)	Ref. [c]
	Amino acids		
6.44	Valine	73-144-75-147-59-74-100-218-145-58	STD
7.24	Proline	73-174-86-147-59-100-75-133-74-175	STD
7.81	Serine	73-57-117-75-101-147-74-219-218-59	STD
8.05	Threonin	73-147-75-55-74-59-133-101-148-233	STD
9.2	Aspartic acid	73-156-174-75-147-86-59-74-100-175	STD
9.26	GABA	73-147-75-220-59-100-218-74-84-55	STD
9.97	Glutamic acid	73-218-75-192-147-74-59-91-58	STD
10.06	Phenylalanine	73-75-103-116-147-74-132-100-59-217	STD
10.4	Asparagine	73-103-147-217-129-74-117-75-133-205	STD
14.06	Tryptophan	73-202-75-131-74-203-116-59-100-55	STD
16.29	Adenosine	73-129-75-103-74-217-147-230-236-55	STD
	Oragnic acid		
7.03	Pyruvate	73-142-75-117-147-59-78-74-180-57	STD
7.21	Nicotinic acid	73-142-75-147-59-143-74-72-66-58	STD
7.34	Succinic acid	73-100-204-218-75-147-59-74-188-116	STD
7.63	Maleic acid	73-147-75-245-100-74-143-59-83-148	STD
8.92	Malonic acid	73-100-147-232-75-74-117-59-156-133	STD
9.13	Salicylic acid	73-267-75-91-135-74-77-149-268-209	STD
11.5	Citric acid	73-103-147-217-75-74-59-89-129-133	STD
	Sugar & Sugar alcohols		
0.37	Xylose	73-116-75-74-132-147-100-141-59-188	STD
10.71	Xylitol	73-103-147-217-129-117-74-75-205-133	STD
11.9	Fructose	73-147-103-160-74-75-129-117-205-217	STD
11.97	Fructose	73-103-147-217-74-75-133-59-117-89	STD
12.22	Glucose	73-202-75-131-74-203-116-59-100-55	STD
12.77	Gluconic acid	73-147-103-74-117-75-217-205-133-129	STD
13.34	myo-inositol	73-147-217-191-103-129-74-133-75-318	STD
16.43	Sucrose	73-103-147-217-129-74-75-169-117-361	STD
16.86	Maltose	73-75-147-103-217-129-74-59-361-204	STD

[a] Retention time; [b] Identified metabolites basend on VIP > 0.7 and a *p*-value < 0.05 from the PLS-DA model; [c] Standard compound.
